# Novel Diffusion Mechanism of Polymers Pinned to an Attractive Impurity

**DOI:** 10.3390/polym14071459

**Published:** 2022-04-03

**Authors:** João C. O. Guerra, Antonio Cadilhe

**Affiliations:** 1 Instituto de Física, Universidade Federal de Uberlândia, Uberlândia 38400-902, Brazil; jcog@ufu.br; 2 Instituto de Física, Universidade Federal da Bahia, Salvador 40210-340, Brazil

**Keywords:** surface contamination, polymer, monte carlo simulations, diffusion

## Abstract

Actual substrates unavoidably possess, to some extent, defects and dirt, which motivate understanding the impact due to their presence. The presence of a substrate naturally breaks symmetries. Additionally, it effectively reduces spatial dimensionality, which favors fluctuation-dominated behavior, but it also provides a multitude of possible interactions. We show evidence of novel behavior in the case of polymer mass transport at a crystalline substrate when a single attractive impurity is present. Specifically, we introduce a model system describing how an attractive impurity pins adsorbed polymers on a substrate. We propose a novel mechanism to explain the size scaling dependence of the diffusion coefficient as $D∼N^{-3/2}$ for polymers with *N* monomers. Additionally, the size dependence of the diffusion coefficient scales can be described as $D∼N^{-\delta }$, with δ=1.51 as determined from extensive simulations.

## 1. Introduction

Challenges involving polymer diffusion, generally speaking, have remained of both fundamental and applied interest. Early studies helped establish bulk diffusion properties on good and poor solvents, along with polymer melts [1,2], while recent studies focused on polymer properties occurring at interfaces [3,4,5,6,7,8,9,10,11,12,13,14,15,16,17]. Models of polymer diffusion act, additionally, as paradigms for other long molecules, e.g., those occurring in biological and biotechnological processes.

The paramount relevance of interfaces and their role over the diffusion of long molecules definitely adds to a deeper, fuller understanding of polymers and life processes, but it requires consideration of more realistic features. For example, real interfaces are inherently contaminated and have defects, kinks, and terraces, which can act as pinning centers to a molecule. In short, if a polymer becomes inserted (or trapped) at an interface, the nature of the latter decisively affects polymer mobility, but a theoretical framework of diffusion on these more realistic environments is presently lacking.

The presence of an interface reduces the effective spatial dimensionality, therefore providing a more controlled environment. After all, many life processes take place by exploiting properties solely arising at an interface, which enhances the survival of an organism. Technological advances frequently take advantage of substrates for added control [18,19]; however, from a theoretical perspective, a reduced spatial dimensionality enhances fluctuations, which could be interpreted as undermining the ability to control. The broader, more complex set of observed phenomena occurring at interfaces favors control through a wide range of specific interactions.

Nevertheless, advances in how polymers and long molecules in general diffuse at interfaces have been consistently accomplished [3,6,7,8,9,10,11,12,13,14,16,17,20,21,22,23,24]. For example, for polymers of *N* monomers (or subunits) diffusing on hard surfaces, the diffusion coefficient scales as $D∼N^{-1}$ and is usually denominated by Rouse regime or Langevin dynamics [6,14,25,26]. Ring polymers have the same size dependence [14], but new issues have been identified [20,27]. In the case of collapsed polymers in poor solvents, the size dependence of the diffusion coefficient decays exponentially [14]. If polymers diffuse in contact with a set of repulsive obstacles, this leads to reptation and to a diffusion coefficient scaling as $D∼N^{-3/2}$ [20]. If hydrodynamic effects are relevant, the size dependence is $∼N^{-1}$ [6,14]. Effects due to confinement, for example, of polymer chains lengths in droplets of various sizes have also been studied [28,29].

In the present work, we introduce a novel system, consisting of an attractive impurity on a substrate pinning a polymer attached to it. Formally, it corresponds to the limit of vanishing substrate contamination, but it can be regarded as the initial condition of common phenomena, where a polymer starts attached to an attractive center, e.g., an impurity or a defect. Novel properties of the model system, to be later justified, involve an intrinsic time-dependence of the diffusion and the presence of anomalous diffusion. Additionally, the size dependence of the diffusion coefficient is

(1) $$D_{N}=D_{o}^{I}N^{-3/2}.$$

These predictions are concomitantly supported by extensive simulations.

In the next section, we start by defining the model system and analyze its properties. In Section 3, we introduce our extensive Monte Carlo simulations, followed by a discussion of the results in Section 4. Finally, in Section 5, we present final remarks.

## 2. Model and Theory

**Figure 1:**
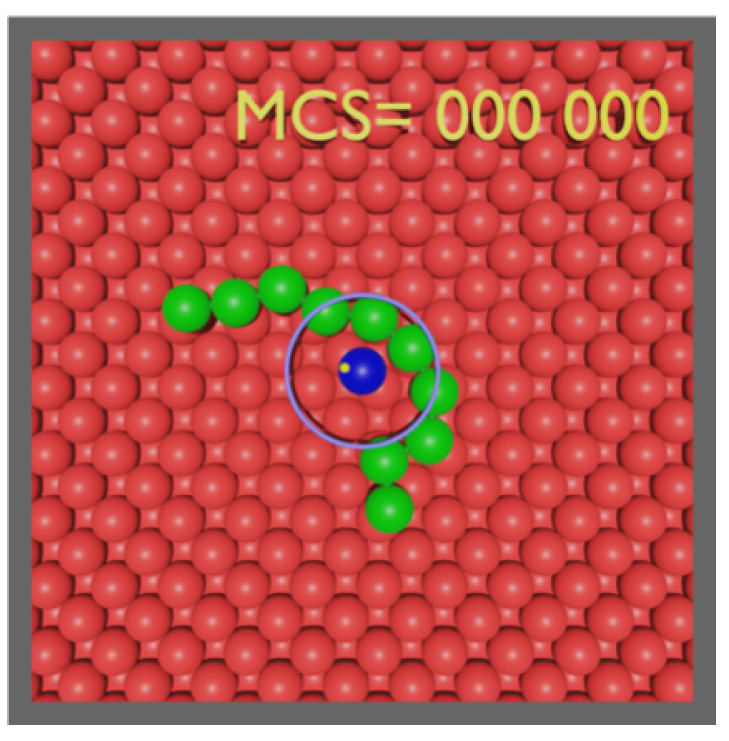
Illustration of a random initial configuration of a ten-monomer polymer (green) adsorbed to an FCC〈100〉 crystal (red) substrate. The midpoint of the segment defined by its central monomers is tangent to a circle of unit radius and centered on the (blue) impurity. The escape, or stop, condition is defined by the midpoint moving past a distance, $r_{c}$, scaling with the size of the polymer, illustrated here by the (purple) ring. The (yellow) dot represents the position of the center of mass of the polymer.

The relevance of the presence of an attractive center to polymer diffusion can be established by the properties of a model system. The model consists of a single attractive impurity placed on a crystalline substrate. The polymer is assumed to be well-adsorbed to the substrate, to prevent detachment from it, where some of its monomers interact with the impurity. Initially, an *N*-monomer polymer is placed, attached to an impurity, on an FCC〈100〉 crystal and let go (see Figure 1). The number of interactions is restricted to a minimum for the sake of simplicity, involving those between monomers, monomers and substrate particles, and monomers and the impurity.

Let us first consider the case of a polymer diffusing on a perfect crystalline substrate without the presence of an impurity. Additionally, the polymer is well-adsorbed to the substrate so that its detachment from the substrate does not occur. It requires sufficiently low temperatures, where monomers jiggle mostly at the bottom of their local energy minima. The motion of a monomer in its basin of attraction is not relevant for polymer diffusion, constituting the so-called fast processes. Polymer diffusion happens whenever a monomer escapes from a local basin of attraction, which represents a thermally activated process with an associated Poisson distribution of escape times. The escape of a monomer from its local basin of attraction requires it to overcome an (effective) energy barrier, $E_{a}^{o}$, just to become trapped on a neighboring one an (equilibrium) distance, $r_{o}$, apart, as imposed by the underlying crystal structure. The motion of the center of mass of the polymer,

(2) $$R_{\mathrm{CM}}=N^{-1}\sum_{n=1}^{N}r_{n},$$

where $r_{n}$ are monomer positions, represents the slowest process, or rate-determining process, as a monomer escape affects its position by a distance of $∼r_{o}/N$.

The diffusion coefficient, *D*, of a polymer is defined through Einstein’s relation,

(3) $$\langle {\left(R_{\mathrm{CM}}\left(t\right)-R_{\mathrm{CM}}\left(0\right)\right)}^{2}\rangle =Dt,$$

where $\langle {\left(R_{\mathrm{CM}}\left(t\right)-R_{\mathrm{CM}}\left(0\right)\right)}^{2}\rangle$ represents the mean square displacement of the polymer center of mass, and *t* is time.

To monitor a polymer diffusing, it becomes convenient to define a constant escape distance, $r_{c}$, while measuring its escape time, $\tau_{e}$. As soon as the polymer moves past such a distance, recording of the evolution of the trajectory is stopped, defining the escape time, $\tau_{e}$, which also represents a first passage time. A consistent reference point from the polymer is required, so the midpoint of the two central monomers of the polymer is utilized to define the escape time. Finally, the distance $r_{c}$ must scale in terms of the radius of gyration,

(4) $$R_{G}^{2}=N^{-1}\sum_{i}^{N}{\left(r_{i}-R_{\mathrm{CM}}\right)}^{2},$$

where $r_{i}$ are the monomer positions in the specific configuration, and $R_{\mathrm{CM}}$ is the position of the center of mass of the polymer. The radius of gyration is a universal quantity, i.e., it depends on a few parameters, such as spatial dimensionality or the presence of excluded volume, scaling as

(5) $$R_{G}∼N^{\nu }.$$

The main assumption of well-adsorbed polymers permits taking ν=3/4, which was obtained from two-dimensional, self-avoiding walks.

To compute the typical timescale involved in the diffusion of a polymer by a distance $r_{o}$, one can consider an ensemble of such systems and calculate the rate, Γ, at which a polymer displaces by $r_{o}$. The same idea also applies to individual monomers belonging to a polymer, say, $\Gamma_{o}$, an escape probability per unit time defined by the lifetime of a Poisson process. Using transition-state theory [21,30,31,32,33,34,35,36,37] permits the calculation of $\Gamma_{o}$, the reciprocal of an escape timescale (from the basin), by relating it to an (effective) vibrational timescale, $\tau_{\mathrm{eff}}$,

(6) $$\Gamma_{o}=\frac{1}{\tau_{\mathrm{eff}}}e^{-\beta E_{a}^{o}},$$

where $\beta =1/k_{B}T$, $k_{B}$ is the Boltzmann constant, and *T* is the temperature. A polymer of size *N* has the diffusion coefficient scaling as

(7) $$D_{N}=\frac{r_{o}^{2}}{\tau_{o}}e^{-\beta E_{a}^{o}}N^{-1}=D_{o}N^{-1},$$

where

(8) $$D_{o}=\frac{r_{o}^{2}}{\tau_{o}}e^{-\beta E_{a}^{o}}.$$

Equation (7) retrieves Rouse or Langevin predictions, which are applied here for thermally activated processes [25,26,38,39]. The basis of the argument to obtain Rouse behavior is the fact that all monomers sample, on average, the same energy landscape and, thus, have $\Gamma =\Gamma_{o}/N$.

The inclusion of an attractive impurity undermines the latter assumption, as its presence implies that few monomers are sampling a different energy landscape from the remaining ones. Eventually, the polymer will escape from its influence, thus returning to Rouse or Langevin behavior. Therefore, its presence does make the system intrinsically time-dependent, which, in turn, removes the equilibrium property of time translation. Hence, ensemble averages of a given quantity, *Q*, are, instead, required at a specified time, *t*, and denoted as 〈Q(t)〉. Since few monomers (possibly none) interact with the impurity at any given time, the majority still diffuse according to Rouse behavior. Nevertheless, the polymer center of mass motion remains as the rate-determining process, but one must now account for the composition of two statistically independent, though concomitant, processes that are responsible for the escaping. One represents the *herd* motion of the *N* monomers, with an energy barrier, $E_{a}^{o}$, resulting in Rouse size dependence of the diffusion coefficient, which can be written as $∼N^{-1}$. The other process involves those few monomers attached to the impurity, which are required to overcome an extra activation energy barrier, $E_{a}^{I}$. Considering that the polymer slides through the impurity along the chain, one monomer at a time, that monomer has an added energy barrier, $E_{a}^{I}$; therefore, it has an overall energy barrier of $E_{a}=E_{a}^{o}+E_{a}^{I}$. Now, regarding the impurity as *moving* in opposite direction along the polymer chain, it samples an energy barrier, $E_{a}^{I}$. Consequently, for the center of mass of the polymer to move by a distance $∼r_{o}$, there must be the above *herd* motion coupled to a *hop* of the impurity along the polymer chain over the same timescale. Now, the impurity performs an isotropic random walk along the polymer chain with a probability

(9) $$p∼\frac{1}{N}e^{-2\beta (E_{a}-E_{a}^{o})}=\frac{1}{N}e^{-2\beta E_{a}^{I}},$$

as the bound monomer is chosen once every *N* attempts, on average. Along the polymer chain, the impurity *moves*, on average, by a distance $r_{o}$, so that

(10) $$D_{N}^{I}=\frac{D_{o}}{N}\sqrt{p}=\frac{D_{o}}{N}\frac{e^{-\beta E_{a}^{I}}}{\sqrt{N}},$$

or

(11) $$D_{N}^{I}=D_{o}^{I}N^{-3/2},$$

where

(12) $$D_{o}^{I}=\frac{r_{o}^{2}}{\tau_{o}}e^{-\beta E_{a}}.$$

Finally, the above result from Equantion (11) corresponds to Equation (1).

## 3. Monte Carlo Simulations

To confirm the above predictions, we performed a set of extensive Metropolis Monte Carlo (MC) simulations, consisting of a set with $2^{14}$ = 16,384 samples for polymers of sizes N=10, 20, 40, 80, and 160, respectively. We found that a temperature of T=1 ensures tightly bound polymers as required by the arguments developed in the previous section.

**Figure 2:**
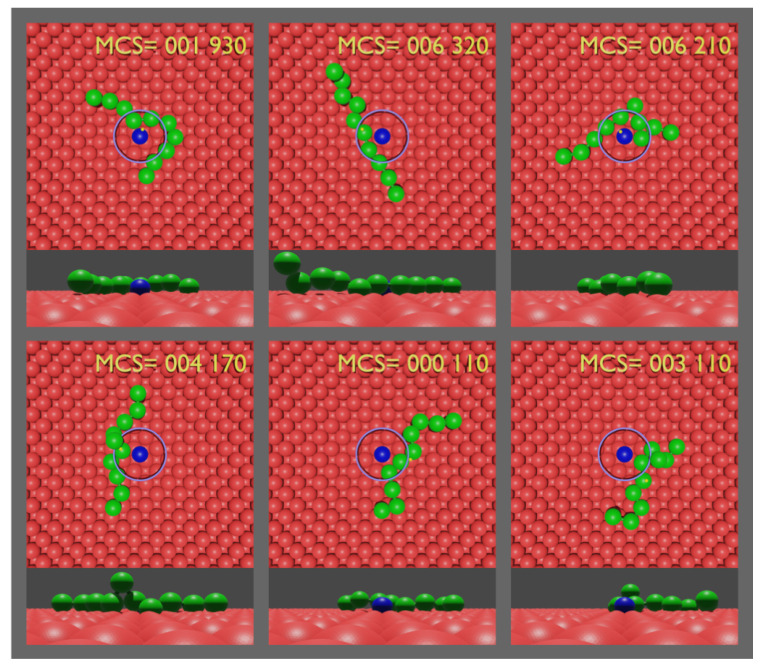
Final configurations of the escape time from various realizations of top and side views of a ten-monomer polymer. The side views represent a *left-to-right* observation of the of the top view, providing support for the requirement of a well-adsorbed polymer. In addition, notice the highly fluctuating escape times of a polymer of the set of samples.

Each sample is initialized with the midpoint of the two central monomers placed at a distance $r_{o}$ from the impurity along a random direction in the plane of the substrate as the example of the initial configuration provided in Figure 1 illustrates. The position of the midpoint of the central monomers on the circle (not shown) of radius $r_{o}$ is randomly chosen (with uniform probability to ensure isotropy) while the monomers are placed tangent to the circle. The simulation of the sample ends when the midpoint moves past a distance $r_{c}=r_{o}+R_{G}/4$ from the impurity (Figure 2). We parametrized the scaling relation for the radius of gyration (Equation (5)) by running a $10^{8}$ MC steps simulation for a ten-monomer polymer, which resulted in $R_{G}=2$.

As the focus is on polymer diffusion, the polymer has to displace by a distance, $r_{c}$, scaling with its size. Therefore, actual short-range details of the potentials are not as relevant to describe the universal behavior related to its diffusion. In this context, the interactions involve two monomer–monomer potentials, a monomer–substrate potential, and a monomer–impurity potential.

Three different pairwise potentials of particle distance *r* were considered, all involving a common length scale, $r_{o}=1$, for the sake of simplicity. Attractive interactions, such as the adsorption of the polymer on the substrate (MS) and binding to the impurity (MI), are modeled by a Lennard–Jones (LJ) potential of different well depths. The LJ potential is defined as $V_{\mathrm{LJ}}\left(r\right)=\epsilon \left(2{\left(r_{o}/r\right)}^{6}-{\left(r_{o}/r\right)}^{12}\right)$, with the MS and MI interactions having different well depths, $\epsilon_{\mathrm{MS}}=-1$ and $\epsilon_{\mathrm{MI}}=-1.5$, respectively.

One of the two monomer–monomer (MM) interactions involves the soft-sphere potential, also known as the Weeks, Chandler, and Anderson potential (WCA) [40]. The WCA potential samples the repulsive part of the LJ potential. This is accomplished by considering the LJ potential up to its minimum, at $r_{o}$, and shift it to zero to solely sample the repulsive part of the potential so that $V_{\mathrm{WCA}}\left(r\right)=V_{\mathrm{LJ}}\left(r\right)+\epsilon$ for $r<r_{o}$ and $V_{\mathrm{WCA}}\left(r\right)=0$ otherwise. The other interaction provides bonding between consecutive monomers utilizing the finite-extent, nonlinear-elastic (FENE) potential [41,42]. The FENE potential is defined as $V_{\mathrm{FENE}}\left(r\right)=-K^{2}r_{o}^{2}/2\ln\left(1-{\left(r-r_{o}\right)}^{2}/r_{o}^{2}\right)$, where $r_{o}$ is the equilibrium distance between consecutive monomers in the chain and K=20 is a constant [42].

## 4. Results and Discussion

The model system has an intrinsic time dependence, as previously discussed, that breaks the time translation property, thus requiring ensemble averages to be performed. Additionally, it is useful to rescale time by defining a maximum observation time, $t_{\max}$, which acts as a cutoff time,

(13) $$\xi =\frac{t}{t_{\max}}.$$

Of course, the value of $t_{\max}$ depends on both the number of monomers of the polymer and the number of remaining (surviving) samples, set to $2^{7}=128$, from a total number of samples of $2^{14}$ = 16,384.

**Figure 3:**
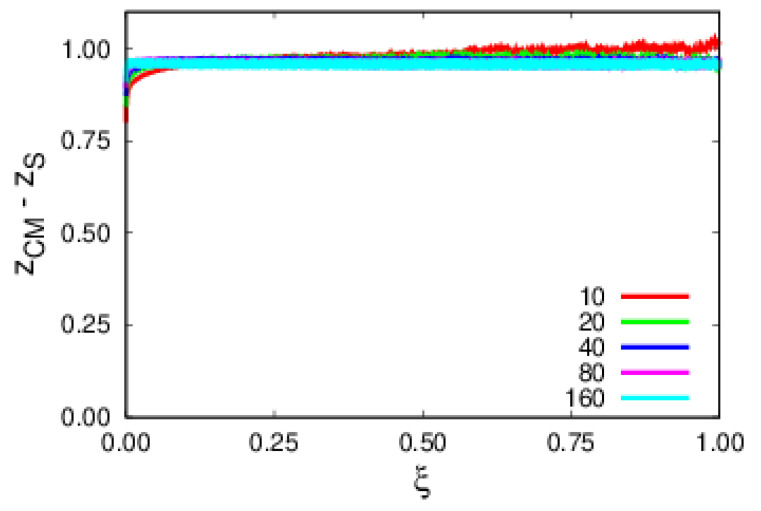
Plots of the position of the *z*-coordinate of the center of mass relative to the top layer substrate for different polymer sizes. In this and the following plots, the sizes and respective colors are N=10 (red), 20 (green), 40 (blue), 80 (purple), and 160 (cyan).

**Figure 4:**
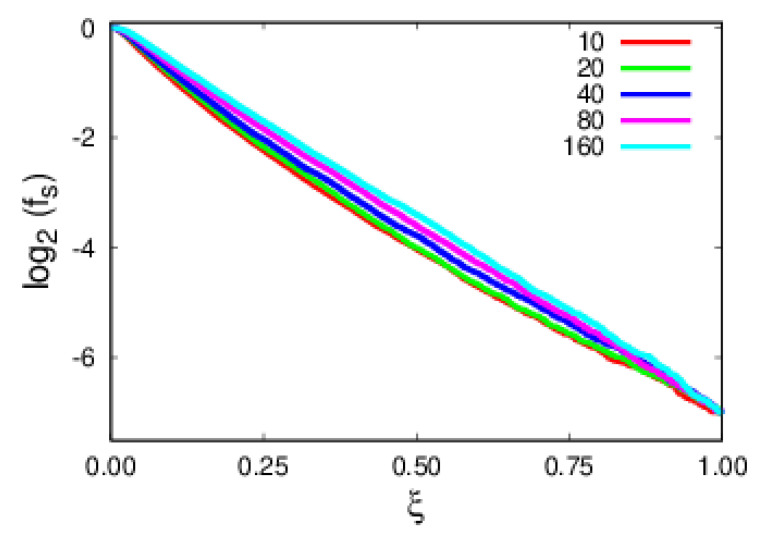
Fraction of surviving samples as a function of rescaled time, ξ, for polymers of various sizes.

Another important aspect of the argumentation was that polymer escapes are thermally activated, hence having a Poisson distribution of escape times. A major assumption of the arguments developed in Section 2 was having polymers well-adsorbed to the substrate. To this end, the height of the *z*-coordinate of the center of mass of the polymer, $Z_{\mathrm{CM}}$, relative to the *z*-coordinate of the top substrate layer, $Z_{S}$, is followed as a function of the rescaled time, ξ. This is fully corroborated by simulations for a temperature of T=1 where it is observed in Figure 3 that polymers remain bound to the substrate at all times. In Figure 4, the log-linear plots of the expected exponential decay in time, ξ, are shown, emphasizing that polymers are thermally activated. A more relevant result is the collapse of the set of polymer sizes into a single curve (Figure 4), revealing the role, in terms of the physics, of rescaled time, ξ.

**Figure 5:**
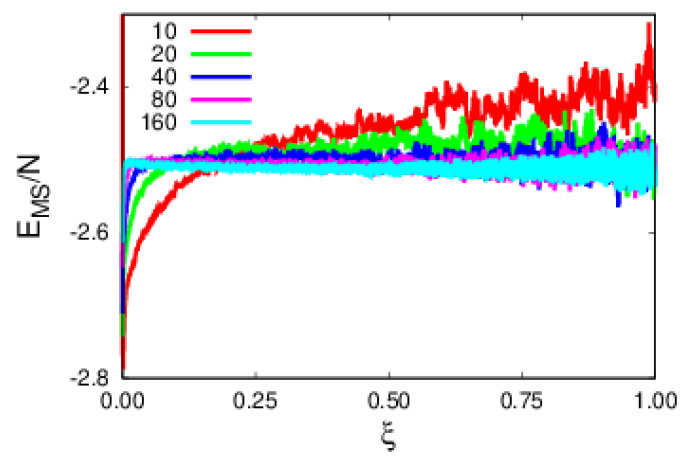
Plots of the monomer–substrate energy contribution per monomer as a function of rescaled time, ξ, for polymers of various sizes.

**Figure 6:**
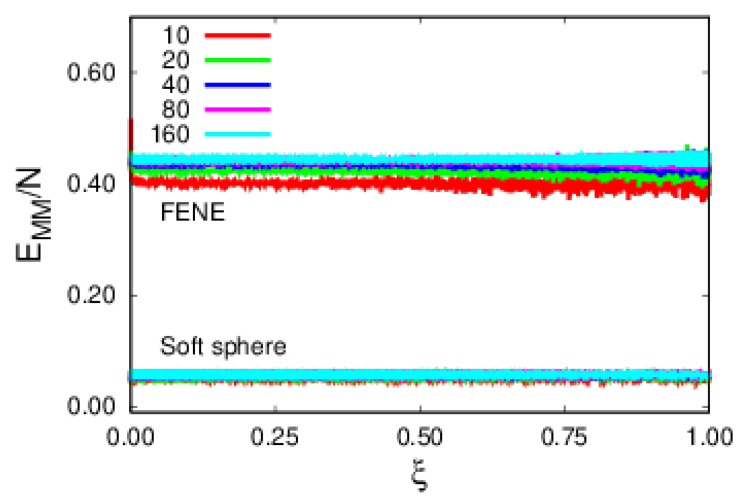
Plots of the two repulsive energy contributions, namely, soft-sphere and FENE, per monomer, as a function of rescaled time, ξ, for polymers of various sizes.

We proceed to the analysis of energy contributions, starting with the monomer–substrate (MS) and monomer–monomer (MM) energies per monomer, as shown in Figure 5. The MS contribution, $E_{\mathrm{MS}}/N$, in Figure 5 decreases with polymer size, therefore implying that larger polymers are more tightly bound to the substrate. Regarding the MM (Figure 6), $E_{\mathrm{MM}}/N$, repulsive contributions, one observes that FENE energy clearly dominates over the soft sphere one. Additionally, FENE shows a size dependence per monomer, while the soft sphere does not.

**Figure 7:**
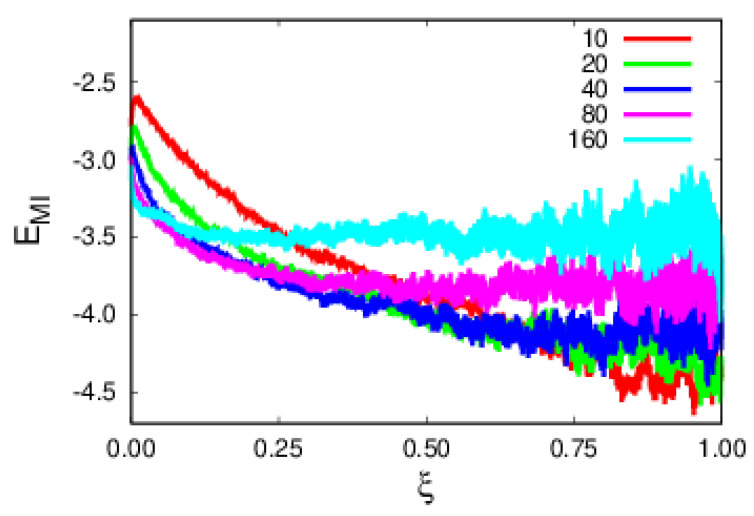
Plots of the monomer–impurity energy contributions in terms of the rescaled time, ξ, for polymers of various sizes.

The argument developed in Section 2 requires few monomers interacting with the impurity. The reason lies on the fact that due to its attractive nature, two monomers (coordination number) become attached to the impurity. Plots of the MI interaction, $E_{\mathrm{MI}}$, in Figure 7 show this behavior, where those interacting monomers have an additional potential well of $\epsilon_{\mathrm{MI}}=-1.5$ (see Section 3). Contrary to the remaining interactions, the monomer–impurity energy has an intrinsic time dependence, as emphasized by the rescaled time plots of Figure 7. The MI binding energy becomes weaker for polymers of larger sizes since these are not as mobile, as evidenced by plots of rescaled time, ξ, even though, as the MI energy varies significantly with polymer size, overall, it does not contribute as much, since it does not scale with the size of the polymer.

**Figure 8:**
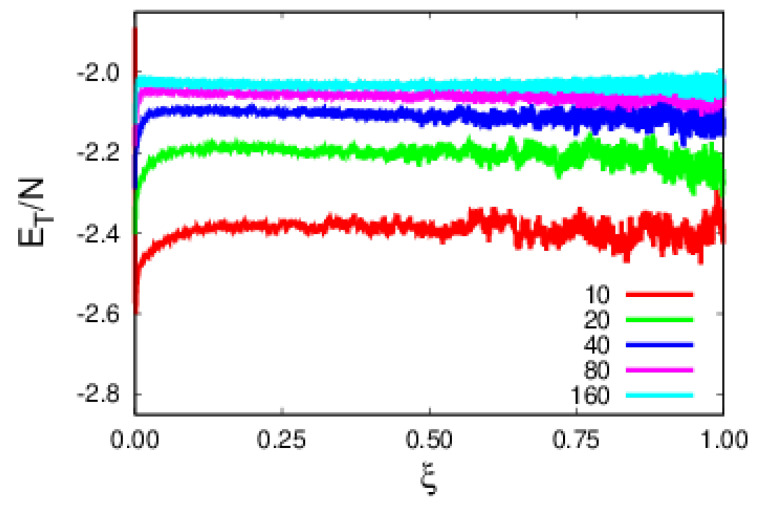
Plots of the total polymer energy per monomer, in terms of ξ for polymers of various sizes.

The total energy per monomer, $E_{T}/N$, shown in Figure 8, has a monotonic increase with polymer size. The observed size dependence is imposed by the stronger size dependence of FENE in relation to MS, as both soft sphere and MI contributions do not have noticeable size dependences.

**Figure 9:**
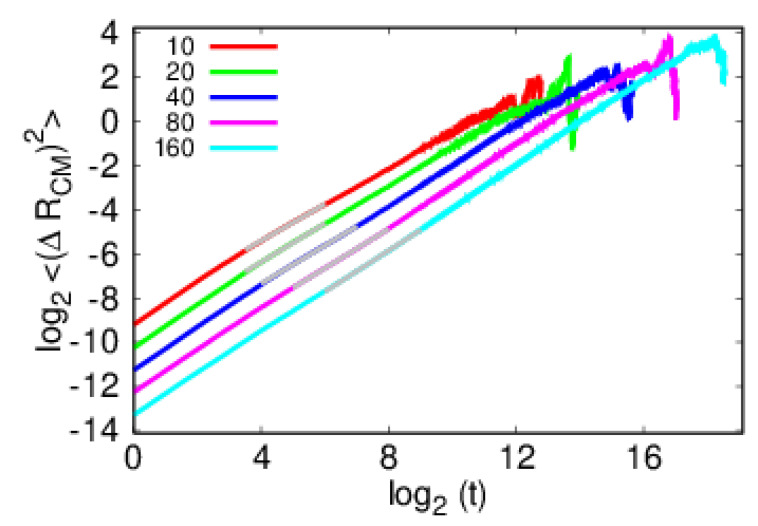
Time dependence of the $\log_{2}$–$\log_{2}$ plots of the mean square displacement of the center of mass relative to its initial position.

The present model has various diffusive regimes, e.g., the initial condition does have to be one of equilibrium, so some adjustment is expected. This transient behavior, present in the MS contribution (Figure 6), does not affect how the polymer detaches from the impurity. After the transient behavior, in the intermediate regime, the polymer still evolves attached to the impurity, as Figure 7 shows, representing the relevant regime of the argument developed in Section 2. Given enough time, the polymer will also escape altogether from its influence, hence crossing over to a regime of diffusion on a clean substrate [6,14,25,39]. In Figure 9 the $\log_{2}$–$\log_{2}$ plots of the time dependence of the mean square displacement of the center of mass of the polymer, $\langle {\left(\Delta R_{\mathrm{CM}}\right)}^{2}\rangle =\langle {\left(R_{\mathrm{CM}}\left(t\right)-R_{\mathrm{CM}}\left(0\right)\right)}^{2}\rangle$, according to Equation (3), show the transient and intermediate regimes.

**Figure 10:**
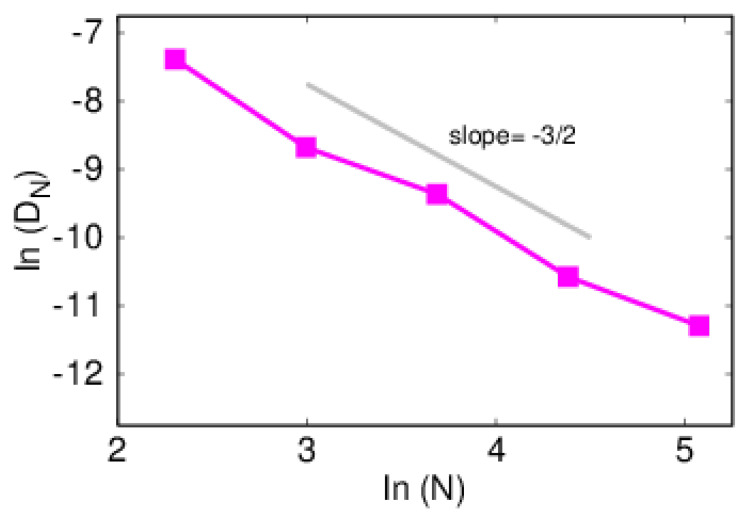
Size dependence of the diffusion coefficient for a polymer initially attached to an impurity. Gray line represents expected slope value.

The size dependence of diffusion coefficient, $D_{N}$, shown in Figure 10, clearly departs from the Rouse behavior of $D∼N^{-\delta }$, with δ=1. In this model, the argument developed in Section 2 predicts a size dependence of δ=3/2, which is corroborated by simulations; the computed value is δ=1.51.

## 5. Final Remarks

We introduced a model system of a polymer initially attached to an impurity site on top a crystalline substrate in order to understand how it diffuses away. In this system, the polymer remains adsorbed to the substrate at all times, and an argument was developed with a predicted size dependence of the diffusion coefficient of ∼$N^{-3/2}$. Furthermore, the prediction was confirmed by extensive simulations by monitoring the motion of the polymer over distances scaling with its size, namely, $r_{c}$, which is related to the gyration radius. The result embodied in Equation (1) is due to a single attractive impurity as opposed to a number of repulsive contact points scaling with the size of the polymer.

The present work raises the need of other studies, such as, for example, the study of polymers attached to a prescribed number of impurity sites, by direct comparison to the present case. In addtion, there is the possibility of researching the implications of finite concentration of contamination.

The result reinforces the need of further studies involving more realistic features of interfaces.

## Data Availability

The data presented in this study are available on request from the corresponding author.
